# Asian Ethnicity is Associated With a Higher Trunk/Peripheral Fat Ratio in Women and Adolescent Girls

**DOI:** 10.2188/jea.JE20110100

**Published:** 2012-03-05

**Authors:** Yukiko Morimoto, Gertraud Maskarinec, Shannon M. Conroy, Unhee Lim, John Shepherd, Rachel Novotny

**Affiliations:** 1 University of Hawaii Cancer Center, Honolulu, HI, USA; 2 University of California San Francisco, San Francisco, CA, USA; 3 University of Hawaii at Manoa, Honolulu, HI, USA; 4 Kaiser Permanente Center for Health Research, Honolulu, HI, USA

**Keywords:** DXA, trunk/peripheral fat ratio, central adiposity, ethnicity

## Abstract

**Background:**

Ethnic differences in body fat mass and distribution may develop in childhood and contribute to increased obesity-related disease risk among Asians. We used dual-energy X-ray absorptiometry (DXA) to evaluate adiposity measures among adult women and their adolescent daughters, who were of predominantly Japanese and white ethnicity.

**Methods:**

We obtained DXA whole body scans for 101 mothers aged 30 years or older and 112 daughters aged 10 to 16 years. The participants were classified as Asian, part-Asian, mixed/other, or white. As a measure of central adiposity, we calculated the trunk/peripheral fat ratio (TPFR). General linear models were used to evaluate differences in adiposity measures by ethnic category.

**Results:**

In mothers, TPFR was significantly higher (*P*_trend_ < 0.01) in Asians and part-Asians (1.38 ± 0.42 and 1.32 ± 0.51) than in mixed/others and whites (1.18 ± 0.27 and 1.09 ± 0.21). The trend was similar among daughters (*P*_trend_ < 0.001), with respective values of 1.09 ± 0.18, 0.97 ± 0.17, 0.99 ± 0.16, and 0.87 ± 0.11. Among mothers, gynoid fat mass and peripheral fat mass were significantly lower in Asians than in whites, whereas none of the regional DXA adiposity measures differed by ethnicity in daughters.

**Conclusions:**

These results confirm previous reports of greater central adiposity in women of Asian ancestry and indicate that ethnic differences in adiposity are already present in adolescence.

## INTRODUCTION

Although obesity is a global health concern, the disease risks associated with obesity differ by ethnicity. As compared with whites, Asians have elevated risks of hypertension, diabetes, and cardiovascular disease at lower body mass index (BMI) values.^1^^–^^3^ This paradoxical finding, which led to evaluation of lower BMI cutoffs for Asians,^4^ also showed the need for measures of adiposity to supplement BMI in assessing the risk of metabolic disease. In particular, studies of Japanese have shown differences as compared with whites in body fat mass and distribution that are not fully captured by BMI alone.^5^^,^^6^ The relatively higher adiposity of Asians as compared with whites is associated with lower birth weight, smaller body frame, and shorter leg to trunk length and therefore reflects the presence of less skeletal and muscle mass and more body and trunk fat for the same BMI, which may be present from childhood.^7^^–^^10^ These characteristics might also be related to the higher proportions of abdominal adiposity, in particular visceral fat, observed in Asian populations.^5^^,^^11^

Dual-energy X-ray absorptiometry (DXA) is used to assess abdominal fat and performs well as compared with computed tomography (CT) and magnetic resonance imaging (MRI) in adults.^12^^,^^13^ In girls of different ethnic backgrounds, DXA-based ratios between trunk and peripheral fat have proven useful in describing ethnic differences.^14^^,^^15^ In this study, to assess whether ethnic differences in body fat mass and distribution develop in early life, we evaluated ethnic differences in trunk-to-peripheral fat ratio (TPFR) and other DXA adiposity measures among adult women and their adolescent daughters of predominantly Japanese and white ethnicity in Hawaii.

## METHODS

### Study design and procedure

The current analysis was conducted as part of a mother–daughter study that used DXA to measure breast density and body-fat composition in adult women and adolescent girls.^16^^–^^18^ The project was approved by the Committee on Human Studies at the University of Hawaii and the Institutional Review Board of Kaiser Permanente (KP) Hawaii, a large health maintenance organization where study participants were recruited. As described in detail elsewhere,^16^^–^^18^ we mailed 3915 invitation letters to women aged 30 years or older who had daughters aged 10 to 16 years; 102 pairs plus 12 additional sisters participated in the study. DXA body scan images were obtained from 101 mothers and 112 daughters for the current analysis.

Before DXA scanning, mothers signed informed consent forms and daughters signed informed assent forms. In addition, all participants answered a questionnaire on demographic and reproductive factors and underwent duplicate height and weight measurement performed by trained research personnel. In mothers, overweight was defined as a BMI of 25 to less than 30 kg/m^2^ and obese as a BMI of 30 kg/m^2^ or higher. In daughters, BMI-for-age percentiles were calculated according to US Centers for Disease Control (CDC) reference data,^19^ and overweight was defined as a BMI in the 85th to less than the 95th percentile and obese as a BMI in the 95th or higher percentile. Tanner stage of breast development was assessed by the same staff member throughout the study.^20^ In the questionnaire, mothers and daughters reported the percentages of all ethnic backgrounds that applied to their parents. Based on this information, each participant was classified as (1) all-Asian; (2) mixed, of partly Asian descent (part-Asian), (3) mixed, of non-Asian descent or other, ie, 1 African American, 1 Hispanic, and 1 Pacific Islander mother and 1 African American daughter (mixed/other), or (4) all-white.

### DXA data collection

At the exam, a urine test excluded pregnancy in all participants. Whole-body scans were performed using the GE Lunar Prodigy Bone Densitometer (GE Healthcare, software version 10.1) to determine body composition, lean soft tissue mass (kg), and fat mass (kg) of standard body regions, including the arms, legs, trunk, and total body. A single DXA operator conducted all DXA scans. The percentage of total body fat was calculated as total fat mass divided by total lean soft tissue mass and total fat mass (all in kg). Scans were reanalyzed for android and gynoid fat mass, using the standardized regions specified by the manufacturer.^21^ Fat mass of the arms and legs was first summed to estimate peripheral fat mass, and TPFR was calculated by dividing trunk fat mass (kg) by peripheral fat mass (kg). Similarly, the android to gynoid fat ratio was calculated as android fat mass (kg) divided by gynoid fat mass (kg).

### Statistical analysis

All statistical analyses were performed using the SAS software package version 9.2 (SAS Institute, Inc., Cary, NC, USA). Anthropometric and DXA adiposity measures across ethnic categories were reported for mothers and daughters as means ± standard deviations. Spearman rank order correlation coefficients were calculated to assess the strength of association between DXA adiposity measures and BMI in both mothers and daughters. General linear models (Proc GLM) were used to evaluate ethnic differences (categorical variable) using log-transformed anthropometric and adiposity data to normalize the distribution; an α of 0.05 was considered significant. In addition, we converted the ethnic categories into a continuous variable in the order of all-white, mixed of non-Asian descent or other, mixed of partly Asian descent, and all-Asian to calculate the *P* value for trend. We also conducted separate analyses that included only 1 of 2 daughters at a time.

## RESULTS

Of the 101 participating mothers (Table 1), 34 were Asian (17 Japanese, 7 Filipino, 5 Chinese, 5 other), 22 part-Asian, 23 mixed/other, and 22 white. Among the 112 daughters (Table 2), the respective numbers were 18 (11 Japanese and 7 other), 47, 30, and 17. Mean ages were 47.0 ± 4.4 years for mothers and 13.9 ± 1.7 years for daughters. Mean BMI was 27.5 ± 6.0 kg/m^2^ for mothers and 21.7 ± 5.0 kg/m^2^ for daughters; age and the proportions of overweight and obese individuals did not differ by ethnic category in either mothers or daughters. Tanner stage did not differ by ethnicity in girls. Percent total DXA body fat was higher in mothers than in daughters (39.8 ± 7.8% vs 30.2 ± 9.1%). In mothers and daughters, the associations between DXA adiposity measures and BMI were strong (Table 3). Among mothers, the correlation coefficients were 0.94 for android fat mass, 0.81 for gynoid fat mass, 0.95 for trunk fat mass, and 0.83 for peripheral fat mass (*P* < 0.0001 for all). The corresponding correlation coefficients for daughters were 0.88, 0.87, 0.86, and 0.82 (*P* < 0.0001 for all). The correlations of BMI with android/gynoid fat ratio and TPFR were weaker than for the fat mass variables: 0.69 and 0.48, respectively, for mothers and 0.68 and 0.49 for daughters (*P* < 0.0001 for all). TPFR values of mothers and daughters were significantly correlated (*r* = 0.29; *P* = 0.002).

**Table 1.** Characteristics of mothers by ethnic category

		Ethnic category				
	All	Asian	Part-Asian	Mixed/other^a^	White	*P* value^b^
*n*	101	34	22	23	22	—
Age (years)	47.0 ± 4.4	48.8 ± 5.7	46.3 ± 4.0	47.2 ± 4.1	47.9 ± 4.3	0.27
Postmenopausal status (*n*)^c^	27	9	4	6	8	0.53
Menopausal hormone use (*n*)	22	11	4	2	5	0.19
Weight (kg)	71.0 ± 17.0	62.8 ± 13.8	72.8 ± 16.6	80.5 ± 17.5	72.2 ± 16.6	<0.001
Height (cm)	160.4 ± 7.3	155.5 ± 5.4	161.1 ± 7.5	163.4 ± 6.1	164.2 ± 6.8	<0.0001
Body mass index (kg/m^2^)	27.5 ± 6.0	26.0 ± 5.7	27.9 ± 5.4	30.1 ± 6.3	26.9 ± 6.1	0.06
Overweight/obese (*n*)^d^	32/28	14/4	5/8	7/10	6/6	0.17

DXA body fat measures						
Total body fat (%)	39.8 ± 7.8	38.1 ± 7.2	39.0 ± 7.3	42.8 ± 8.0	40.0 ± 8.7	0.23
Android fat (kg)	2.6 ± 1.44	2.3 ± 1.3	2.6 ± 1.3	3.2 ± 1.6	2.5 ± 1.5	0.17
Gynoid fat (kg)	5.3 ± 1.92	4.4 ± 1.4	5.2 ± 1.6	6.4 ± 1.9	5.7 ± 2.3	0.0001
Android/gynoid fat ratio	0.48 ± 0.16	0.52 ± 0.18	0.50 ± 0.17	0.47 ± 0.14	0.41 ± 0.11	0.08
Trunk fat (kg)	15.0 ± 13.4	13.1 ± 6.0	15.3 ± 6.9	17.9 ± 7.8	14.6 ± 7.2	0.11
Peripheral (arm and leg) fat (kg)	12.8 ± 10.8	9.5 ± 3.5	11.8 ± 4.4	14.9 ± 4.7	13.1 ± 5.0	<0.0001
Trunk/peripheral fat ratio	1.26 ± 0.39	1.38 ± 0.42	1.32 ± 0.51	1.18 ± 0.27	1.09 ± 0.21	0.02

Abbreviation: DXA, dual-energy X-ray absorptiometry.

^a^Mixed/other includes 1 African American, 1 Hispanic, and 1 Pacific Islander.

^b^*P* values for anthropometric and DXA measures are based on log-transformed data and reflect overall difference across ethnicity as a categorical variable.

^c^Postmenopausal status was defined as no menstrual period for ≥365.25 days; data were missing for 1 white woman.

^d^Based on body mass index: overweight, 25 to <30; obese, ≥30.

**Table 2.** Characteristics of daughters by ethnic category

			Ethnic category				
		All	Asian	Part-Asian	Mixed/other^a^	White	*P* value^b^
*n*		112	18	47	30	17	—
Age (years)		13.9 ± 1.7	14.4 ± 1.6	13.5 ± 1.9	14.3 ± 1.6	13.8 ± 1.5	0.14
Tanner breast stage	1–2	17	1	10	4	2	0.33
	3	38	10	15	7	6	
	4–5	57	7	22	19	9	
Weight (kg)		53.9 ± 14.5	50.7 ± 12.1	55.1 ± 16.8	54.8 ± 13.3	52.6 ± 11.8	0.75
Height (cm)		157.4 ± 8.4	154.9 ± 6.7	156.3 ± 9.6	159.2 ± 7.3	159.9 ± 7.9	0.12
BMI (kg/m^2^)		21.7 ± 5.0	21.4 ± 4.1	22.3 ± 5.5	21.5 ± 4.5	20.7 ± 5.4	0.62
BMI z-score		0.40 ± 1.02	0.28 ± 1.07	0.57 ± 1.07	0.36 ± 0.86	0.10 ± 1.10	0.39
Overweight/obese (*n*)^c^		15/15	3/2	8/8	3/3	0/2	0.53

DXA body fat measures							
Total body fat (%)		30.2 ± 9.1	28.1 ± 8.4	30.4 ± 9.4	31.0 ± 9.4	30.2 ± 8.8	0.75
Android fat (kg)		1.3 ± 1.0	1.1 ± 0.6	1.3 ± 1.1	1.3 ± 1.0	1.1 ± 0.7	0.91
Gynoid fat (kg)		3.5 ± 1.5	3.1 ± 1.2	3.5 ± 1.7	3.6 ± 1.5	3.3 ± 1.3	0.81
Android/gynoid fat ratio		0.33 ± 0.10	0.35 ± 0.09	0.34 ± 0.11	0.33 ± 0.11	0.30 ± 0.09	0.51
Trunk fat (kg)		7.9 ± 5.0	7.1 ± 3.9	8.2 ± 5.6	8.3 ± 5.1	7.1 ± 4.2	0.81
Periphery (arm and leg) fat (kg)		7.9 ± 4.3	6.5 ± 3.4	8.2 ± 4.9	8.1 ± 4.2	7.9 ± 3.6	0.43
Trunk/periphery fat ratio		0.98 ± 0.17	1.09 ± 0.18	0.97 ± 0.17	0.99 ± 0.16	0.87 ± 0.11	**0.001**

Abbreviations: BMI, body mass index; DXA, dual-energy X-ray absorptiometry.

^a^Mixed/other includes 1 African American.

^b^*P* values for anthropometric and DXA measures are based on log-transformed data and reflect overall difference across ethnicity as a categorical variable.

^c^Based on BMI-for-age percentile: overweight, 85 to <95th percentile; obese ≥95th percentile.

**Table 3.** Correlation of DXA adiposity measures with BMI in mothers and daughters^a^

		% Total body fat	Android fat (kg)	Gynoid fat (kg)	Android: gynoid fat ratio	Trunk fat (kg)	Peripheral (arm and leg) fat (kg)	Trunk: peripheral fat ratio
Mothers (*n* = 101)	*r*	0.85	0.94	0.81	0.69	0.95	0.83	0.48
	*P* value	<0.0001	<0.0001	<0.0001	<0.0001	<0.0001	<0.0001	<0.0001

Daughters (*n* = 112)	*r*	0.79	0.88	0.87	0.68	0.86	0.82	0.49
	*P* value	<0.0001	<0.0001	<0.0001	<0.0001	<0.0001	<0.0001	<0.0001

^a^Spearman rank order correlation coefficients.

Android fat mass, android/gynoid fat ratio, and trunk fat mass did not differ by ethnicity in mothers; however, gynoid fat mass and peripheral fat mass were lower among Asian groups as compared with non-Asian groups (*P* = 0.0001 and *P* < 0.0001; Table 1). Due to lower peripheral fat mass, TPFR was significantly higher in Asian groups than in non-Asian groups (Figure): 1.38 ± 0.42 for Asians and 1.32 ± 0.51 for part-Asians vs 1.18 ± 0.27 for mixed/other and 1.09 ± 0.21 for whites (*P*_trend_ < 0.01 on significant trend test). Mean TPFR among individuals with 100% Japanese ancestry (*n* = 17) was very similar to that for all-Asians (1.38 ± 0.50). In daughters, TPFR was the only DXA body fat measure that differed by ethnicity (Table 2); the linear decline with decreasing degree of Asian admixture (*P*_trend_ < 0.001) was similar to that seen in mothers (Figure). The respective mean TPFR values were 1.09 ± 0.18, 0.97 ± 0.17, 0.99 ± 0.16, and 0.87 ± 0.11. Mean TPFR of 100% Japanese daughters (*n* = 10) was 1.08 ± 0.17. All results were similar in analyses that included only 1 of 2 siblings for the 12 participants with sisters. After stratification by weight status, mean TPFR was 1.09 ± 0.28 for normal-weight mothers and 1.37 ± 0.41 for overweight/obese mothers (*P* < 0.0001); the respective values for daughters were 0.95 ± 0.16 and 1.07 ± 0.15 (*P* < 0.001).

**Figure.**
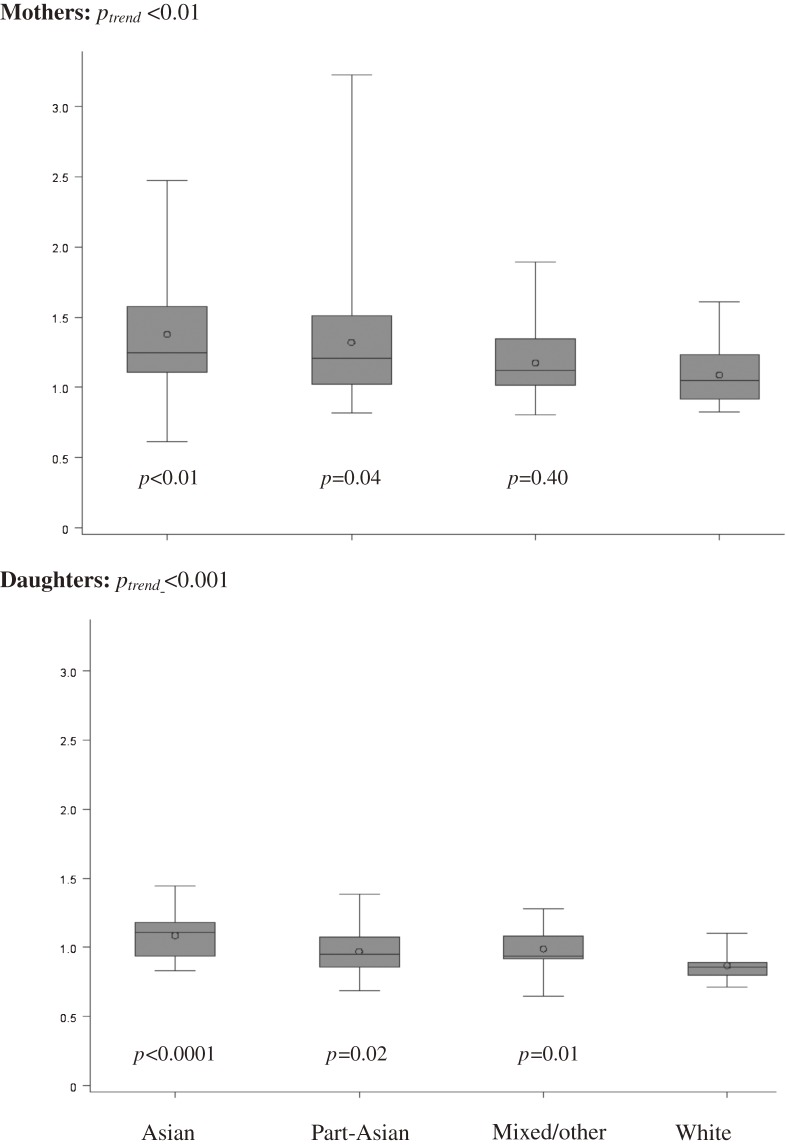
Distributions of trunk/peripheral fat ratio in mothers (top) and daughters (bottom) by ethnic category; *P* values are based on log-transformed data, with whites as reference, and *P*_trend_ values are generated using ethnicity as a continuous variable in the order of white, mixed/other, part-Asian, and Asian. The horizontal lines from the bottom to the top correspond to the 10th, 25th, 50th (median), 75th, and 90th percentiles, and the circle represents the arithmetic mean.

## DISCUSSION

We observed ethnic differences in DXA adiposity measures among women and girls of predominantly Asian and white ethnicity in Hawaii. As compared with non-Asian mothers and their adolescent daughters, those of Asian ancestry had a higher TPFR, which strongly suggests that Asian ethnicity is associated with higher central adiposity, as previously observed by other studies and measures,^6^^,^^22^^,^^23^ and that these ethnic differences begin early in life, as was reported in a study of Indian infants.^24^ Although the high overall correlations of DXA adiposity measures with BMI confirm the validity of BMI as a universal obesity measure, lower correlations with TPFR and android/gynoid fat ratio indicate that % body fat and adipose tissue distribution are not precisely predicted by BMI. Thus, central adiposity measures are important in evaluating the risks of obesity-related disease across populations that have varying adiposity patterns.^11^ According to our results, TPFR may be useful in comparing central adiposity across ethnically diverse populations.

These results agree with previous comparisons in girls^14^ and support the hypothesis that ethnic differences in adipose tissue distribution arise in childhood, possibly due to ethnic variations in skeletal dimensions^14^^,^^21^ or perinatal events.^10^^,^^24^ TPFR appears to reflect the ethnic variations in skeletal dimensions more closely than does the android/gynoid fat ratio. It may thus be a more precise estimate of central adiposity and provide more information than the android/gynoid fat ratio among younger populations. A recent study showed that a DXA measure of trunk-to-extremity fat ratio which was similar to TPFR was better than MRI as an indicator of visceral adiposity in normal-weight and obese girls.^15^ Ethnic differences in trunk-to-limb fat mass ratio exist between white, African-American, and Hispanic adults,^25^^,^^26^ and reference values are available from the National Health and Nutrition Examination Survey (NHANES) for these groups^26^ but not for Asians and youths. Similarly, NHANES provides information on lean and fat mass and other body composition data for non-Asian children and adolescents.^27^^,^^28^

A major strength of the present study is the inclusion of adult women and adolescent girls, in particular those of Asian and mixed-Asian ethnicity for whom data are limited, and the detailed assessment of ethnicity. A number of limitations should also be noted. Due to the small sample size and low response rate of the study, the observed ethnic differences need to be confirmed in a larger study. Furthermore, stratified analyses of normal-weight and overweight/obese women should be included in future investigations, as should analyses of men, who have different body compositions and larger abdominal diameters than women.^26^ An important limitation of DXA is its inability to distinguish subcutaneous from visceral fat mass, which can be differentiated by CT and MRI.^12^ Because visceral adiposity has been found to be more strongly associated than subcutaneous fat with diabetes and other chronic conditions,^29^^,^^30^ MRI and CT have been used in some research studies.^23^^,^^31^ However, these methods are not feasible for use in population-based studies or screening settings due to their high cost and the relatively high radiation exposure associated with CT. In contrast, DXA is more practical due to its low cost, is available in clinical settings, and results in minimal radiation exposure. The relatively weak correlation of DXA with BMI and the difference in TPFR by BMI status in mothers suggests that DXA provides information about body fat distribution that BMI does not. Nevertheless, as indicated by the low participation rate, the DXA method is primarily a research instrument to understand fat distribution across ethnic groups and not a tool for clinical assessment. To evaluate their use as screening tools, additional evaluations of TPFR and other DXA adiposity measures are needed. In addition, enhanced DXA methods capable of distinguishing subcutaneous from visceral fat are under development.

This innovative study confirmed the higher prevalence of trunk adiposity among women and girls of primarily Japanese ancestry, as was observed in other populations,^6^^,^^23^ and provides evidence that these differences are present during adolescence, which makes a genetic, epigenetic, or growth-related origin likely. Given the increase in obesity-related metabolic disease among Japanese and other Asians,^1^^–^^3^ a better understanding of ethnic differences in body fat composition and distribution might provide critical information for developing ethnic-specific, preventive measures for these conditions.

## References

[1] Stommel M, Schoenborn CA Variations in BMI and prevalence of health risks in diverse racial and ethnic populations. Obesity (Silver Spring). 2010;18:1821–6 10.1038/oby.2009.472 20075855

[2] Maskarinec G, Erber E, Grandinetti A, Verheus M, Oum R, Hopping BN, Diabetes incidence based on linkages with health plans: the multiethnic cohort. Diabetes. 2009;58:1732–8 10.2337/db08-1685 19258435 PMC2712787

[3] Huxley R, James WP, Barzi F, Patel JV, Lear SA, Suriyawongpaisal P, Ethnic comparisons of the cross-sectional relationships between measures of body size with diabetes and hypertension. Obes Rev. 2008;9 Suppl 1:53–61 10.1111/j.1467-789X.2007.00439.x 18307700

[4] WHO Expert Consultation Appropriate body-mass index for Asian populations and its implications for policy and intervention strategies. Lancet. 2004;363:157–63 10.1016/S0140-6736(03)15268-3 14726171

[5] Hayashi T, Boyko EJ, McNeely MJ, Leonetti DL, Kahn SE, Fujimoto WY Visceral adiposity, not abdominal subcutaneous fat area, is associated with an increase in future insulin resistance in Japanese Americans. Diabetes. 2008;57:1269–75 10.2337/db07-1378 18299316

[6] Kadowaki T, Sekikawa A, Murata K, Maegawa H, Takamiya T, Okamura T, Japanese men have larger areas of visceral adipose tissue than Caucasian men in the same levels of waist circumference in a population-based study. Int J Obes (Lond). 2006;30:1163–5 10.1038/sj.ijo.0803248 16446744

[7] Deurenberg P, Deurenberg-Yap M, Guricci S Asians are different from Caucasians and from each other in their body mass index/body fat per cent relationship. Obes Rev. 2002;3:141–6 10.1046/j.1467-789X.2002.00065.x 12164465

[8] Deurenberg P, Deurenberg-Yap M, Wang J, Lin FP, Schmidt G The impact of body build on the relationship between body mass index and percent body fat. Int J Obes Relat Metab Disord. 1999;23:537–42 10.1038/sj.ijo.0800868 10375058

[9] Deurenberg P, Deurenberg-Yap M, Foo LF, Schmidt G, Wang J Differences in body composition between Singapore Chinese, Beijing Chinese and Dutch children. Eur J Clin Nutr. 2003;57:405–9 10.1038/sj.ejcn.1601569 12627175

[10] Yajnik CS Early life origins of insulin resistance and type 2 diabetes in India and other Asian countries. J Nutr. 2004;134:205–10 14704320 10.1093/jn/134.1.205

[11] Lear SA, Humphries KH, Kohli S, Chockalingam A, Frohlich JJ, Birmingham CL Visceral adipose tissue accumulation differs according to ethnic background: results of the Multicultural Community Health Assessment Trial (M-CHAT). Am J Clin Nutr. 2007;86:353–9 17684205 10.1093/ajcn/86.2.353

[12] Snijder MB, Visser M, Dekker JM, Seidell JC, Fuerst T, Tylavsky F, The prediction of visceral fat by dual-energy X-ray absorptiometry in the elderly: a comparison with computed tomography and anthropometry. Int J Obes Relat Metab Disord. 2002;26:984–93 12080454 10.1038/sj.ijo.0801968

[13] Kamel EG, McNeill G, Han TS, Smith FW, Avenell A, Davidson L, Measurement of abdominal fat by magnetic resonance imaging, dual-energy X-ray absorptiometry and anthropometry in non-obese men and women. Int J Obes Relat Metab Disord. 1999;23:686–92 10.1038/sj.ijo.0800904 10454101

[14] Novotny R, Daida YG, Grove JS, Le Marchand L, Vijayadeva V Asian adolescents have a higher trunk:peripheral fat ratio than Whites. J Nutr. 2006;136:642–7 16484537 10.1093/jn/136.3.642 PMC1478165

[15] Savgan-Gurol E, Bredella M, Russell M, Mendes N, Klibanski A, Misra M Waist to hip ratio and trunk to extremity fat (DXA) are better surrogates for IMCL and for visceral fat respectively than for subcutaneous fat in adolescent girls. Nutr Metab (Lond). 2010;7:86 10.1186/1743-7075-7-86 21143876 PMC3018385

[16] Maskarinec G, Morimoto Y, Daida Y, Laidevant A, Malkov S, Shepherd JA, Comparison of breast density measured by dual energy X-ray absorptiometry with mammographic density among adult women in Hawaii. Cancer Epidemiol. 2011;35:188–93 10.1016/j.canep.2010.06.009 20688593 PMC3081054

[17] Maskarinec G, Morimoto Y, Daida Y, Shepherd J, Novotny R A comparison of breast density measures between mothers and adolescent daughters. BMC Cancer. 2011;11:330 10.1186/1471-2407-11-330 21810248 PMC3161041

[18] Novotny R, Daida Y, Morimoto Y, Shepherd J, Maskarinec G Puberty, body fat, and breast density in girls of several ethnic groups. Am J Hum Biol. 2011;23:359–65 10.1002/ajhb.21145 21445936

[19] Centers for Disease Control and Prevention/National Center for Health Statistics. CDC Growth Charts. Available from: http://www.cdc.gov/growthcharts August 4, 2009. Accessed June 22, 2010.

[20] Tanner JM. Growth at adolescence, with a general consideration of the effects of hereditary and environmental factors upon growth and maturation from birth to maturity. 2nd ed. Oxford: Blackwell Scientific Publisher; 1962.

[21] Novotny R, Going S, Teegarden D, Van LM, McCabe G, McCabe L, Hispanic and Asian pubertal girls have higher android/gynoid fat ratio than whites. Obesity (Silver Spring). 2007;15:1565–70 10.1038/oby.2007.185 17557994

[22] Tanaka S, Horimai C, Katsukawa F Ethnic differences in abdominal visceral fat accumulation between Japanese, African-Americans, and Caucasians: a meta-analysis. Acta Diabetol. 2003;40 Suppl 1:S302–4 10.1007/s00592-003-0093-z 14618500

[23] Lim U, Ernst T, Buchthal SD, Latch M, Albright CL, Wilkens LR, Asian women have greater abdominal and visceral adiposity than white women with similar body mass index. Nutr Diabetes. 2011;1:published online 9 May 2011 10.1038/nutd.2011.2 PMC3302135 23449381

[24] Yajnik CS, Lubree HG, Rege SS, Naik SS, Deshpande JA, Deshpande SS, Adiposity and hyperinsulinemia in Indians are present at birth. J Clin Endocrinol Metab. 2002;87:5575–80 10.1210/jc.2002-020434 12466355

[25] Rahman M, Temple JR, Breitkopf CR, Berenson AB Racial differences in body fat distribution among reproductive-aged women. Metabolism. 2009;58:1329–37 10.1016/j.metabol.2009.04.017 19501860 PMC2728780

[26] Kelly TL, Wilson KE, Heymsfield SB Dual energy X-Ray absorptiometry body composition reference values from NHANES. PLoS ONE. 2009;4:e7038 10.1371/journal.pone.0007038 19753111 PMC2737140

[27] Borrud LG, Flegal KM, Looker AC, Everhart JE, Harris TB, Shepherd JA Body composition data for individuals 8 years of age and older: U.S. population, 1999–2004. Vital Health Stat 11. 2010;1–87 20812448 PMC5901781

[28] Flegal KM, Ogden CL, Yanovski JA, Freedman DS, Shepherd JA, Graubard BI, High adiposity and high body mass index-for-age in US children and adolescents overall and by race-ethnic group. Am J Clin Nutr. 2010;91:1020–6 10.3945/ajcn.2009.28589 20164313 PMC2844683

[29] Fox CS, Massaro JM, Hoffmann U, Pou KM, Maurovich-Horvat P, Liu CY, Abdominal visceral and subcutaneous adipose tissue compartments: association with metabolic risk factors in the Framingham Heart Study. Circulation. 2007;116:39–48 10.1161/CIRCULATIONAHA.106.675355 17576866

[30] Liu J, Fox CS, Hickson DA, May WD, Hairston KG, Carr JJ, Impact of abdominal visceral and subcutaneous adipose tissue on cardiometabolic risk factors: the Jackson Heart Study. J Clin Endocrinol Metab. 2010;95:5419–26 10.1210/jc.2010-1378 20843952 PMC2999970

[31] Park YW, Allison DB, Heymsfield SB, Gallagher D Larger amounts of visceral adipose tissue in Asian Americans. Obes Res. 2001;9:381–7 10.1038/oby.2001.49 11445659

